# Manual lymphatic drainage and quality of life in patients with lymphoedema and mixed oedema: a systematic review of randomised controlled trials

**DOI:** 10.1007/s11136-018-1796-5

**Published:** 2018-02-05

**Authors:** Martin Müller, Karsten Klingberg, Maria M. Wertli, Helena Carreira

**Affiliations:** 1 Department of Emergency Medicine, Inselspital, Bern University Hospital, University of Bern, Freiburgstrasse, 3010 Bern, Switzerland; 2 Division of General Internal Medicine, Inselspital, Bern University Hospital, University of Bern, Bern, Switzerland; 3 Department of Non-Communicable Disease Epidemiology, Faculty of Epidemiology and Population Health, London School of Hygiene & Tropical Medicine, London, United Kingdom

**Keywords:** Quality of life, Musculoskeletal manipulations, Edema, Review, Systematic

## Abstract

**Purpose:**

To assess the impact of manual lymphatic drainage (MLD) on the health-related quality of life (HRQoL) of adults with lymphoedema or mixed oedema, through a systematic review of randomised controlled trials (RCTs).

**Methods:**

MEDLINE, EMBASE, PsycINFO, CENTRAL, the Cochrane Database of Systematic Reviews and ClinicalTrials.gov were searched to identify RCTs evaluating HRQoL after a MLD intervention compared to non-MLD interventions (PROSPERO 2016:CRD42016042255). We extracted the effect of the interventions on the HRQoL (primary outcome) as well as data on volume and functional changes, and adverse events when available (secondary outcomes).

**Results:**

Eight studies were eligible. The studies were heterogeneous in the aetiology of oedema, schemes of MLD applied, additional treatments offered with MLD, length of follow-up, instruments used to assess HRQoL and interventions offered to the control group. Five studies included patients with breast cancer-related arm lymphoedema; one study reported increased HRQoL among patients randomised to the MLD group. The two RCTs that involved patients with leg mixed oedema due to chronic venous insufficiency did not find between-group differences in the overall HRQoL. One trial included patients with hand oedema from systemic sclerosis and showed higher HRQoL in the group that received MLD. No studies reported reductions in HRQoL, or severe adverse events after MLD. The small numbers of patients analysed in all studies may have resulted in lack of power to detect between-group differences in HRQoL.

**Conclusions:**

The effect of MLD on the HRQoL of patients with chronic oedema is unclear.

**Electronic supplementary material:**

The online version of this article (10.1007/s11136-018-1796-5) contains supplementary material, which is available to authorized users.

## Introduction

Lymphoedema is a chronic abnormal swelling of a limb or quadrant of the trunk due to accumulation of protein-rich fluids in the interstitial tissue, caused by incapacity of the lymphatic system to effectively distribute lymph [1, 2]. Most lymphoedema cases are secondary to cancer treatments such as lymphadenectomies and/or radiotherapy [3, 4]. Breast cancer patients are particularly affected, with nearly 20% of the women who have axillary lymph node dissection developing lymphoedema of the arm [5, 6].

Mixed oedema, also called lymphaticovenous oedema, is a form of chronic oedema that results from damages to the lymphatic system caused by prolonged volume overload from venous diseases, such as post-thrombotic syndrome or chronic venous insufficiency [7–9].

Chronic oedema may have severe physical and psychological consequences for patients, including impaired physical function, recurrent infections, ulcerations, pain, limb numbness, heaviness and tightness, as well as reduced quality of life [10–13]. Guidelines for the management of breast cancer-related lymphoedema (BCRL) recommend the use of complete decongestive therapy (CDT) [1, 2], which includes manual lymphatic drainage (MLD), self-care (e.g. healthy diet, skin care), physical exercise and compression therapy with bandaging or garments [14]. CDT is also used in the management of mixed oedema [15, 16]. MLD consists of special massage techniques with gentle tissue pressure to promote lymph flow [14]. It is considered effective in reducing lymphoedema [17]. Nevertheless, besides understanding the impact of MLD on clinical endpoints (e.g. changes in volume or appearance of ulceration), understanding the MLD impact on patients’ perception, including the health-related quality of life (HRQoL) is essential to develop comprehensive therapy concepts.

To the best of our knowledge, no systematic review aimed to evaluate the effect of MLD on the HRQoL of patients with lymphoedema or mixed oedema. A systematic review that aimed to assess the efficacy and safety of MLD to treat BCRL included two trials that also evaluated quality of life; however, none of the trials presented results for between-group comparisons  and thus the effect of MLD on HRQoL was inconclusive [18]. A systematic review on the quality of life of patients with cancer-related lower limb lymphoedema suggested that CDT may improve HRQoL, but this conclusion was based on two small observational studies [15].

We aimed to evaluate the impact of MLD on the HRQoL of adult patients with lymphoedema or mixed oedema, irrespective of the oedema aetiology or location, through a systematic review of randomised controlled trials (RCTs).

## Methods

The review protocol was registered at the international prospective register of systematic reviews (PROSPERO 2016:CRD42016042255).

### Search strategy

We searched MEDLINE, EMBASE and PsycINFO via OvidSP®, and the Cochrane Central Register of Controlled Trials (CENTRAL), the Cochrane Database of Systematic Reviews and ClinicalTrials.gov, from inception up to June 2016. The search expressions are provided in Online Appendix S1. In addition, the reference lists of the eligible studies and of the systematic reviews were manually screened to identify additional studies.

### Criteria for considering studies for this systematic review

RCTs including patients with lymphoedema or mixed oedema, in which MLD or CDT (exposure) was the primary intervention given to the intervention group (IG) and not to the control group (CG), and providing results for HRQoL, were eligible. There were no restrictions in the aetiology of oedema, affected body region, or duration or frequency of the MLD intervention. We considered as eligible studies involving patients with post-thrombotic syndrome because mixed oedema is part of the natural course of the disease [19–21]. Studies involving patients with chronic venous insufficiency in the grade of oedema (> C2, as measured by the Clinical-Etiologic-Anatomic-Pathophysiologic score [22]) were also eligible.

The following exclusion criteria were defined a priori and applied: observational study design; studies not including adults (< 18 years); RCTs that did not include patients with lymphoedema or mixed oedema; RCTs with a primary intervention other than MLD; RCTs in which the CG received MLD or a MLD-like intervention (e.g. self-lymphatic drainage or automatic-MLD performed by technical devices, as these interventions may attenuate the between-group differences); RCTs not providing measurements of HRQoL (overall or domains) before and after the intervention in both groups and RCTs not providing sufficient data for a comparison of the primary outcome data in CG and IG. No language criterion was applied.

### Selection of studies

Two reviewers (MM, KS) independently screened the references, applying the inclusion and exclusion criteria (see above). The studies were evaluated in two steps: first considering the information provided in the title and abstract, and then the full-text. When the violation of a criterion could not be determined unequivocally, the article was considered for further evaluation.

Differences in the decision of the two reviewers were solved by discussion and involving a third researcher (HC) when necessary.

### Data extraction

The following data were extracted using a pre-defined form: characteristics of the studies (e.g. RCT design; data to evaluate the risk of bias); characteristics of the study population (e.g. age at trial enrolment, sex distribution, aetiology of the oedema and affected body part); characteristics of the intervention (e.g. duration and frequency of the MLD intervention and additional interventions) and outcome data (e.g. mean or median scores of HRQoL summary measures, or of HRQoL domains, as available, and results of hypothesis tests). When a trial reported results for more than one follow-up time, we extracted the data for the first follow-up after the MLD intervention; this aimed to reduce between-trial variability in the time elapsed since the intervention and the evaluation of the outcome.

In one study with three treatment groups, the two groups in which the interventions differed only in the MLD-treatment were included in the analysis [23]. In one cross-over trial, only first cycle data were extracted to avoid carry-over and period effects [24].

### Risk of bias

The risk of bias was assessed based on the domains proposed by the Cochrane Collaboration’s for intervention studies [24]. We sought information to evaluate the potential for selection bias due to lack of randomisation and allocation concealment. Patients cannot be blinded to the MLD intervention; however, we kept this item in our assessment to highlight the potential for detection bias.

Bias could also have occurred in the trials if the adherence to, or the quality of MLD intervention, were suboptimal. The risk of bias due to lack of adherence to MLD was considered low when patients in the IG attended at least 75% of the initially planned number of MLD sessions, high when patients attended less than 75% of the sessions and unclear when the number of attended sessions was not reported [18]. A low risk in quality of MLD satisfies the property that skilled therapists performed the MLD intervention.

Information on the quality of the studies was used to comment on the results.

### Data synthesis

The primary outcome was overall HRQoL, as defined by the authors of the original studies. Overall HRQoL and HRQoL domains are often measured with several psychometric scales. The HRQoL domains from the conceptual framework of the Short Form Health Survey 36 [25], a widely used and accepted HRQoL questionnaire, were used to group individual domains reported by the different HRQoL instruments. Therefore, we summarised data under the following domains of HRQoL: physical, social, psychological/mental, role functioning, body pain, vitality and general.

Descriptive tables were used to summarise the mean/median scores between the IG and the CG (between-group differences), as well as the pre- and post-intervention mean/median scores within each group. Study results are described by oedema aetiology and affected body region.

The statistical significance level of the between-group differences is for the comparisons of the post-intervention HRQoL measure, or the mean difference of the treatment effect (pre-post value), between IG and CG, as reported in the original studies.

Follow-up time, defined as the time elapsed between the last MLD-treatment and the HRQoL assessment, was grouped into three categories: (1) immediate follow-up, if HRQoL was measured between 1 day and 2 weeks following the last treatment; (2) short-term follow-up, if between 2 weeks and 12 weeks; (3) intermediate-term follow-up, if more than 12 weeks.

Meta-analytic methods were not used due to the heterogeneity of the eligible trials regarding the study populations, LE definition, interventions offered to intervention and control groups, instruments used to quantify HRQoL and time elapsed between MLD and assessment of HRQoL.

## Results

**Fig. 1:**
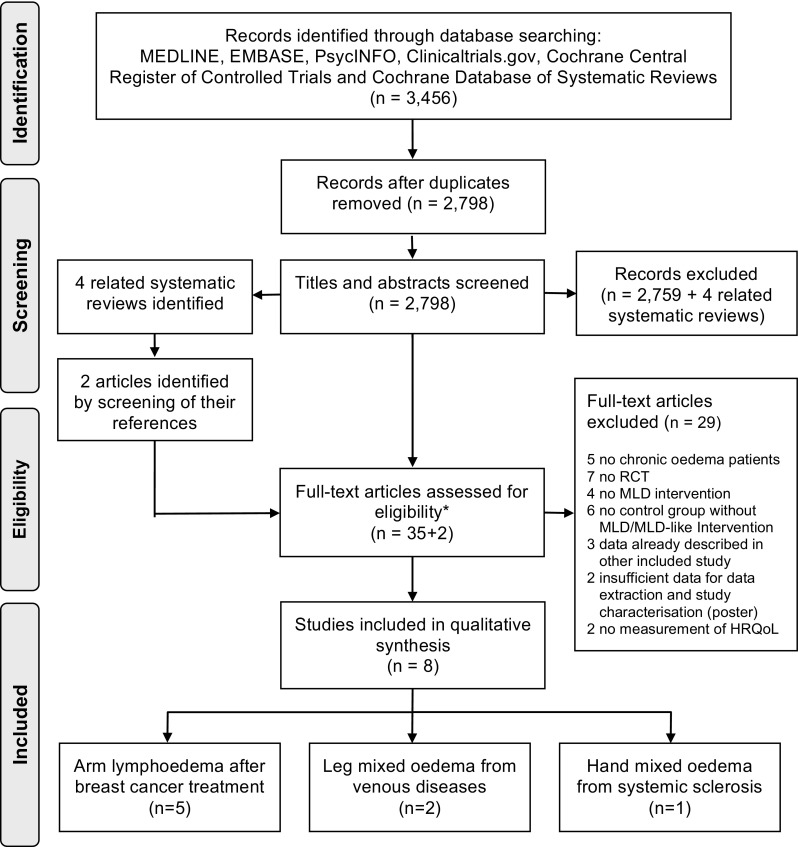
Flowchart of the systematic review process. *HRQoL* health-related quality of life, *MLD* manual lymphatic drainage, *RCT* randomised controlled trial. *No additional studies were identified through screening of the references of the included articles

Out of 3456 references identified in the electronic databases, 37 full-text papers were assessed for eligibility and eight [23, 26–32] were included in the analysis (Fig. 1).

In the eight eligible studies, three study populations, areas and aetiologies of lymphoedema were identified: arm BCRL (five studies [23, 26, 28, 29, 31]), mixed oedema from venous diseases in the legs (two studies [30, 32]) and hand oedema from systemic sclerosis (1 study [27]) (Table 1).

**Table 1:** Main characteristics of the studies included in the systematic review

First author (year of publication)	Country	RCT design	Aetiology, area with oedema	Definition of oedema	Sample size IG // CG		Age^a^ in IG // CG	% men IG // CG	Intervention				Timeliness of follow-up^b^	Tool used to evaluate overall HRQoL
					Recruited	Analysed			Treatment	Duration and frequency of MLD	Additional interv.(s)	Control-group intervention(s)		
Belmonte (2012) [26]	Spain	Cross-over	BCR, arm	Clinical diagnosis, according the ISL consensus	19//17	18//14	70 ± 10 // 66 ± 13	0 // 0	CDT	50 min, 5x/w, 2w	CP garments, advice on skin care, exercise	Low-frequency low-intensity electrotherapy	Immediate	FACT-B + 4
Dayes (2013) [28]	Canada	Parallel group	BCR, arm	10% volume difference of the limbs (affected vs. non-affected)	57 // 46	56 // 39	61 (36–86) // 59 (41–76)	0 // 0	CDT	60 min 5x/w, 4w	Elastic CP garments, advice on skin care, exercise	Elastic CP garments, advice on skin care, exercise	Immediate	
Gradalski (2015) [29]	Poland	Parallel group	BCR, arm	Stage-II lymphoedema, according to the ISL (≥ 20% difference between limb volumes)	30 // 30	25 // 26	61 ± 9 // 62 ± 12	0 // 0	CDT	30 min, 5x/w, 2w	CP bandaging and physical exercise	CP bandaging and physical exercise	Short-term	QoL-LQ
Odebiyi (2014) [31]	Nigeria	Parallel group	BCR, arm	Arm-swelling without further characterisation	n.r. // n.r.	17 // 10	46 ± 8 // 54 ± 14	0 // 0	MLD 1	5 min, 2x/w, 6w	Physical exercise	Physical exercise	Intermediate	EORTC QLQ-C30
Ridner (2013) [32]	United States	Parallel group^3^	BCR, arm	Stage I or II lymphoedema, according the ISL (1995)	n.r. // n.r	16 // 15	68 ± 10 // 66 ± 11	0 // 0	MLD	40 min, n.r., mean 10 sessions^a^	Low-level laser therapy	Low-level laser therapy	Immediate	ULL-27 FACT-G FACT-B
Bongi (2011) [27]	Italy	Parallel group^c^	SSc, hand	Clinical diagnosis	20 // 20	20 // 15	57 ± 10 // 57 ± 13	0 // 0	MLD	60 min,1x/w, 5w	–	Observation	Immediate	HAQ
Holmes (2014) [30]	United States	Parallel group	PTS, leg	Clinical diagnosis	Not specified	15 // 16	47 (27–66) // 49 (22–82)	47 // 38	CDT	n.r, mean of 12 in 12w	Skin care, CP bandaging, physical exercise, education	CP bandaging	Immediate	VEINES-QOL
dos Santos Crisostomo (2015) [33]	Portugal	Parallel group	VI, leg	CVI with a CEAP score of C3 to C5	25 // 25	20 // 21	55 ± 11 // 47 ± 11	25 // 5	MLD	45 min, 10x in 4w	Educational session	Educational session	Immediate and short-term	CIVIQ-20

*BCR* breast cancer-related, *CDT* complete decongestive therapy, *CEAP* clinical-etiologic-anatomic-pathophysiologic classification, *CIVIQ-20* Chronic Venous Insufficiency Quality-of-Life Questionnaire—20 items, CG control group, CP compression, CVI chronic venous insufficiency, EORTC QLQ-C30 European Organisation for Research and Treatment of Cancer Quality-of-Life Questionnaire Core 30, *FACT-B, FACT-G, FACT-S* functional assessment of cancer therapy(-breast)(-general)(-subscale), *HAQ* Health Assessment Questionnaire, *IG* intervention group, *ISL* International Society of Lymphology, *MLD* manual lymphatic drainage, *n. r*. not reported, *PTS* post-thrombotic syndrome, *QoL-LQ* Quality-of-Life Lymphoedema Questionnaire, *RCT* Randomised Controlled Trial, *SSc* systemic sclerosis, *ULL-27* upper limb lymphoedema-27, *VEINES-QOL* Venous Insufficiency Epidemiological and Economic Study Quality-of-Life questionnaire, *w* weeks

^a^Mean ± standard deviation or mean (range)

^b^Immediate follow-up: 1 day to 2 weeks following the last treatment; short-term follow-up: more than 2–12 weeks; intermediate- term follow-up: more than twelve weeks to 1 year; long-term follow-up: more than 1 year

^c^Three-arm randomised controlled trial

### Risk of bias

**Fig. 2:**
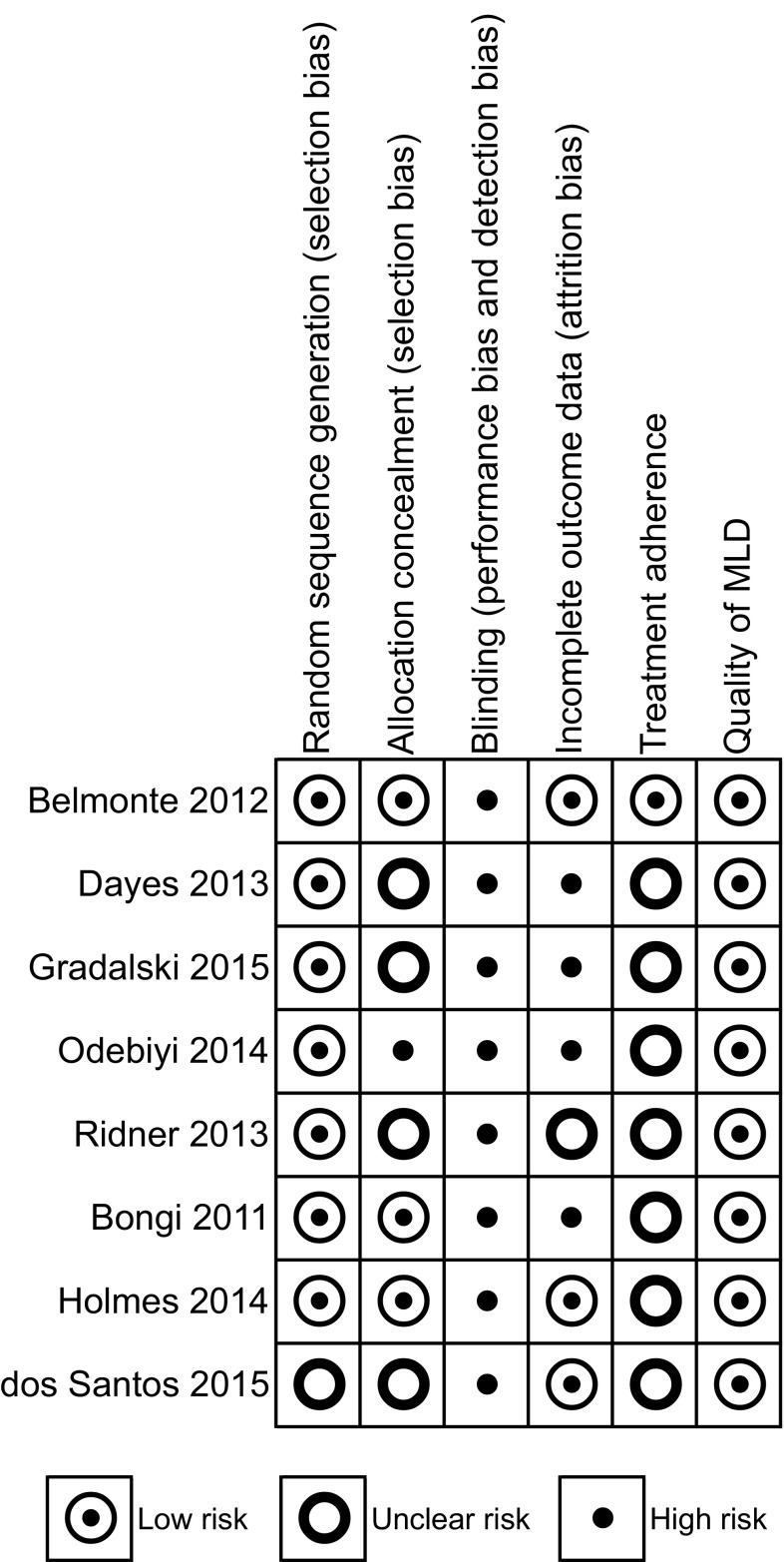
Risk of bias summary. Footnotes: blinding of the patients could not be done due to the nature of the intervention and outcome. The selective reporting category judges the evidence of incomplete reporting of outcomes, time points, subgroups or analyses. Treatment adherence measures whether the prescribed therapy sessions were completed as planned. Quality of MLD categorises the risk of bias due to an incorrect/insufficient MLD intervention (e.g. when applied by unskilled therapists). *MLD* manual lymphatic drainage; *HRQoL* health-related quality of life

The risk of bias due to unbalanced probability of group allocation was low in most studies. However, in one study the computer-generated random list was not concealed [31] and this information was unclear in four others [23, 28, 29, 32]. Performance bias was judged high for all trials because the physical nature of the intervention precludes the blinding of the patients to the exposure. The risk of attrition bias was considered high in four studies [27–29, 31] due to imbalances in the number of withdrawals that could be related to the outcome between the groups (Fig. 2).

The baseline characteristics did not show any significant differences between IG and CG, except for the study [28] where patients in the IG suffered from more severe lymphoedema.

### Breast cancer-related lymphoedema

Five studies included women who developed lymphoedema after breast cancer treatments [23, 26, 28, 29, 31]. The studies used different criteria to define lymphoedema, including clinical diagnosis [26, 31] as well as measures of limb volume difference [23, 28, 29]. All trials included few patients in analysis, ranging from 16 [23] to 56 [28] in the IG and from 10 [31] to 39 [28] in the CG.

Heterogeneity was also observed in the interventions offered in addition to MLD to the IGs and to the CGs. CDT was the primary intervention in three studies [26, 28, 29]; other concomitant interventions were exercise [31] and low-level laser therapy [23]. The schemes of MDL-sessions used in the trials varied from 15 min, twice a week, over 6 weeks [31] to 60 min, five times a week, during 4 weeks [28]. Control interventions consisted of exercise, CDT without MLD, electrotherapy and laser therapy (Table 1).

All but one study assessed HRQoL with a breast cancer- or lymphoedema-specific questionnaire (Online Appendix S2). Odebiyi et al. [31] used a generic instrument for cancer patients (the European Organisation for Research and Treatment of Cancer quality-of-life questionnaire). The domains covered by each instrument are provided in Online Appendix S3. Most trials used instruments that included domains for physical and psychological functioning [23, 26, 28, 29] and pain [26, 29]. The first outcome evaluation was mostly performed immediately after the last MLD-treatment [23, 26, 28] (Table 1).

Table 2 summarises the main results of the comparison of overall HRQoL and HRQoL domains between the intervention and control group, as well as the comparison of pre- and post-intervention. Statistical evidence for improved HRQoL in the group offered MLD was found in one study only (MLD and physical exercise vs. physical exercise) [31] (Table 2). In the study by Gradalski et al. [29], the overall HRQoL did not differ between the two groups, but the group offered MLD reported significant improvements in social functioning.

**Table 2:** Summary of the statistical significance of the differences in overall HRQoL and HRQoL domains between the intervention and control group, and pre- and post-intervention in the intervention group

First author (year of publication)	Area of oedema	HRQoL															
		Overall		Physical		Role		Mental		Social		Pain^a^		Vitality		General	
		BG	PP	BG	PP	BG	PP	BG	PP	BG	PP	BG	PP	BG	PP	BG	PP
Belmonte (2012) [26]^b^	Arm	○	–	▲/○^f^	–	○	–	○	–	○	–	○	–			–	–
Dayes (2013) [28]	Arm	–	–	○	○	–	–	○	○	–	–	–	–	–	–	–	–
Gradalski (2015) [29]	Arm	○	▲	○	○/▲^c^	○	○/▲^d^	○	○/▲^g^	○/▲^h^	○/▲^i^	○	▲	–	–	–	–
Odebiyi (2014) [31]	Arm	▲	▲	–	–	–	–	–	–	–	–	–	–	▲	▲	–	–
Ridner (2013) [32]	Arm	○	–	○	–	○	–	○	–	○	–	–	–	○	–	○	–
Bongi (2011) [27]	Hand	▲	▲	▲	▲	▲	▲	▲	▲	–	–	▲	▲	–	–	–	
Holmes (2014) [30]	Leg	○	○	○	○	–	–	–	–	–	–	○	○	–	–	–	–
dos Santos Crisostomo (2015) [33]	Leg	○	–	○/▲^e^	▲^e^	–	–	○	–	○	–	▲	▲	▲	▲	–	–

*BG* between-group difference, *CG* control group, *HRQoL* health-related quality of life, *IG* intervention group, *PP* pre- vs. post-intervention mean in the IG

“▲”,evidence against the null hypothesis of no difference between the treatment groups in the outcome (*p* < 0.05), i.e. there is evidence that there is a benefit of MLD concerning the outcome

“○”, no evidence against the null hypothesis (*p* ≥ 0.05)

^a^Of the affected body part

^b^Cross-over trial: treatment effect in the whole sample (both) groups

^c^For the symptom of limb heaviness, not for movement restriction

^d^For issues with clothing, not for interference with work, interference household or dependence on others

^e^For the symptom of heaviness, not in the used physical functioning subscale of the HRQoL score

^f^Improvements in tightness but not in heaviness or in the physical well-being subscale

^g^Improvements in well-being and subjective limb appearance, but not in sleep disturbances

^h^Improvements in interference with social life and hobbies but no interference with social relationships

^i^Improvements in interference with intimate relationships but not with social life or hobbies

Within each group, overall HRQoL generally improved from pre- to post-intervention (Online Appendix S3); some statistical evidence of within group improvements was reported by Gradalski et al. (CDT vs. CDT without MLD) [29], Odebiyi et al. (MLD and physical exercise vs. physical exercise) [31] and Ridner et al. (MLD and low-level laser therapy vs. low-level laser therapy) [23], both for the intervention and control groups (Table 2). Gradalski et al. [29] also reported significant improvements in well-being feelings, lymphoedema-related pain, limb heaviness and size, skin tension, in addition to less sleep disturbances and skin infections, both in the intervention and in the control group, even though there were no statistically significant differences between the two groups (Online Appendix S3).

Almost all trials reported lymphoedema and upper limb volume measurements (Table 3). A reduction in lymphoedema volume pre- and post-intervention was noted in all trials [23, 26, 28, 29]. However, only Dayes et al. [28] reported a significant reduction in the absolute lymphoedema volume in the intervention group, compared to the control group.

**Table 3:** Adverse events and secondary outcomes reported by the trials: comparison between intervention and control groups (BG) and pre- and post-treatment in the intervention group (PP)

First author (year of publication)	Adverse events		Oedema volume		Volume affected body part		Functional outcomes	
	IG	CG	BG	PP	BG	PP	BG	PP
Belmonte (2012) [26]^a^	None	1 erysipelas	○	▲	–	–	–	–
Dayes (2013) [28]	19 events^b,c^	9 events^c,d^	Absolute:▲	▲	–	–	–	–
			Relative:○	–				
Gradalski (2015) [29]	n.r.	n.r.	○	▲	○	▲	–	–
Odebiyi (2014) [31]	n.r.	n.r.	–	–	▲	▲	–	–
Ridner (2013) [32] n.r	n.r.	○	▲	○	▲			
Bongi (2011) [27]	None	n.r.	▲	▲	▲	▲	▲	▲
Holmes (2014) [30]	1 deep venous thrombosis	2 deep venous thrombosis, 1 thrombophlebitis, 1 stocking allergy	–	–	–	–	–	–
dos Santos Crisostomo (2015) [33]	n.r.	n.r.	–	–	○	–	○	

*BG* between-group difference, *CG* control group, *DVT* deep venous thrombosis, *HRQoL* health-related quality of life, *IG* intervention group, *MLD* manual lymphatic drainage, *n.r*. not reported (unable to determine if assessed), *PP* pre- vs. post-intervention difference in the outcomes reported by the IG

“▲”, statistical evidence against the null hypothesis of no difference between the treatment groups in the outcome (*p* < 0.05), i.e. there is evidence that there is a benefit of MLD concerning the outcome

“○”, no evidence against the null hypothesis (*p* ≥ 0.05)

^a^Cross-over trial: treatment effects/adverse events in the whole sample (both) groups

^b^In 17 patients

^c^Most events consisted of temporary rash or mild to moderate pain. One episode of cellulitis and severe pain occurred in the IG

^d^In 7 patients

Adverse events were rarely reported. The few cases reported included temporary rash, pain and one episode each of cellulitis [28] and erysipelas [26] (Table 3).

### Leg mixed oedema

Two studies included patients diagnosed with mixed oedema from venous diseases [30, 32]. Holmes et al. [30] included 31 patients with post-thrombotic syndrome and evaluated the effect of MLD as part of CDT compared to compression bandaging alone. Dos Santos Crisostomo et al. [32] analysed data for 41 patients with chronic venous insufficiency, to compare the effect of ten sessions (45 min) of MLD over four weeks and an educational session, to that of the educational session alone. The evaluation of the outcome was performed immediately after the last intervention in both studies, using disease-specific instruments that include items for oedema (Table 1). Overall HRQoL was not significantly different between the intervention and control group in neither study. Significant differences in HRQoL were, however, reported for patients with post-thrombotic syndrome receiving either therapy but who did not wear stockings before the study, compared to those who wore the stockings [30] (Table 2). Further a posteriori analyses revealed that those with more severe post-thrombotic syndrome had greater improvements in HRQoL after either treatment, but these results were not statistically significant. Within group differences pre- and post-intervention were only reported for pain and fatigue by patients with chronic venous insufficiency who received MLD and an educational session [32] (Table 2).

Adverse events included three cases of deep venous thrombosis (IG: *n* = 1; CG *n* = 2) [30], and one case each of superficial thrombophlebitis and stocking allergy in the compression garments only group [30] (Table 3).

### Hand oedema in patients with systematic sclerosis

One study assessed the effect of MLD on the HRQoL of 20 patients with systemic sclerosis and clinically diagnosed hand oedema, compared to observation alone (control group, *n* = 20) [27] (Table 1). The results showed significant improvements in HRQoL in the IG (one hour session of MLD per week, over 5 weeks) compared to the placebo CG [27]. The difference in overall HRQoL was accompanied by between-group differences in the physical, mental, role functioning and pain domains [27] (Table 2). Significant changes in HRQoL between baseline and the end of treatment were reported in the IG only, for overall HRQoL as well as for the physical and mental components [27]. The volume of the hand and of oedema reduced significantly after the MLD intervention, and the volume post-MLD was significantly lower when compared to the women in the CG [27]. No adverse events were reported [27].

## Discussion

### Summary of main results

We found conflicting evidence of the impact of MLD on the HRQoL of adult patients with lymphoedema or mixed oedema. Most studies showed that MLD did not significantly increase the HRQoL of the patients with BCRL or mixed oedema due to venous diseases. However, significant increases in HRQoL or some of its domains were reported in patients with systemic sclerosis and hand oedema, and in one out of the five studies that included patients with BCRL. The studies were heterogeneous in regard to the definition of lymphoedema, interventions offered to the intervention and control groups across trials and the time points at which the HRQoL was evaluated. In summary, the quantity, quality and heterogeneity of the trials preclude definitive conclusions.

### Comparison with other studies

Breast cancer patients are one of the largest groups of lymphoedema patients. CDT, MLD and bandaging have been shown to be effective in reducing lymphoedema volume [15], which was also described in the trials included in this review [28, 29]. A previous systematic review reported inconclusive results on the effect of MLD in the quality of life of patients with BCRL [18]. Our study includes four studies published since then [23, 28, 29, 31], most of which report no changes in HRQoL in comparison to the control groups. Lack of improvement in HRQoL after MLD interventions was also found in studies that were not eligible for this review because they included self-lymphatic drainage in the control group [33] or were non-randomised trials [34]. Breast cancer patients seem to have relatively high levels of HRQoL at baseline [23], which might affect the extent to which the overall HRQoL can be improved. One uncontrolled study, however, reported significant improvements in HRQoL after CDT compared to the control intervention [35].

Improvements were reported for certain HRQoL domains such as pain, heaviness, emotional function, dyspnoea and sleep disturbance [33] and should not be overlooked. Even though no study reported decreases in HRQoL after MLD interventions, lack of power to detect a negative association cannot be ruled out.

Non-pharmacological therapeutic options for oedema from chronic venous insufficiency include compression garments, movement and MLD [36]; the latter along the route of the venous vessels was shown to increase the blood flow in the deep and superficial veins [37]. Improvements in HRQoL were only described for patients who did not use stockings before the intervention. We excluded two studies of patients with chronic venous insufficiency because both patients with and without oedema were included; these were placebo-controlled trials that reported significant improvements in HRQoL after MLD [12, 38]. While these results from placebo-controlled trials are promising, more trials with multimodal therapy programs are needed to quantify the relative contribution of MLD in CDT [18].

Systemic sclerosis is a rare but highly debilitating disease for which treatment options are scarce. The impact of any intervention on the HRQoL of the patients is of the utmost importance but MLD has been hardly ever studied [39]. The results reported here must be interpreted with caution, as they come from a single study.

The mechanisms through which MLD might lead to higher HRQoL are diverse but common to all patients. The pressure on the tissues reduces the microlymphatic hypertension and stretches the lymph collector, increasing the lymphatic transport capacity, which results in decreased volume of the affected body part. Volume decrease may reduce discomfort (e.g. pain, tightness and heaviness) as well as improve the function of the affected body region [18]. Besides the effect on lymphatic vessels, the blood flow in superficial arteries and veins increases, improving wound healing and decreasing inflammatory makers [14]. Additionally, MLD is usually combined with skin care, exercise, which have a positive effect on the HRQoL [40], and the role of social interaction and relaxation should not be disregarded [14].

### Study limitations

The small number of eligible studies limits this review, as in some areas/aetiology of oedema only one or two studies were identified, and therefore conclusions must be drawn with caution. We searched six well-known databases, as well as the references of systematic reviews and of studies included, with the aim of identifying an exhaustive list of references. It is unlikely that important trials on the topic were missed.

Another important limitation comes from the small sample size of the eligible trials. This is particularly relevant because our results relied on the *p* values reported by the studies to take into account chance as an alternative explanation for their results, and some trials may not have had statistical power to detect a significant effect of the MLD on HRQoL, should one exist.

Direct comparisons among the studies were also limited by the different HRQoL instruments used, which contained items for different domains. Disease- and condition-specific questionnaires do not always measure the same domains, and generic HRQoL scales might not capture the symptoms of lymphoedema sufficiently.

In most studies MLD was offered in combination with physical exercise, skin care, compressive garments or other innovative interventions. These interventions may have a positive impact in the HRQoL of the patients, and therefore the effect reported in the trials may not be directly comparable to trials that offered MLD only.

### Implications for practice and future research

In the absence of evidence showing that MLD has an adverse effect in HRQoL, doctors and therapists should emphasise the benefits in volume reduction and potential positive effects on some HRQoL domains to their patients (e.g. for vitality [31]).

More high-quality studies, in patients with oedema at different locations and of different severity, are needed to provide a definitive answer to this question. If MLD proves to have a positive effect on the HRQoL of patients with chronic oedema, more research is needed to find an optimal treatment duration and frequency, and to quantify the relative contribution of the treatment effect of MLD in multimodal therapy concepts [1, 17].

## Conclusions

In conclusion, the effect of MLD on the HRQoL of the patients with chronic oedema is unclear. However, MLD is a well-tolerated, accepted and safe treatment technique with shown benefits for oedema volume reduction that should be continued in the absence of evidence for a negative impact on the HRQoL of the patients.

## Electronic supplementary material

Below is the link to the electronic supplementary material.

Supplementary material 1 (PDF 86 KB)

Supplementary material 2 (PDF 71 KB)

Supplementary material 3 (PDF 107 KB)
