# Supplementary material for: Manual lymphatic drainage and quality of life in patients with lymphoedema and mixed oedema: a systematic review of randomised controlled trials
Source: Qual Life Res. 2018 Feb 5;27(6):1403–14. doi: 10.1007/s11136-018-1796-5 (PMC5951867; doi:10.1007/s11136-018-1796-5)
Supplement: Supplementary file 1 — Supplementary material 1 (PDF 86 KB) [file 11136_2018_1796_MOESM1_ESM.pdf]

Appendix S1. Search strategies

MEDLINE search expression

#	Searches	
1	exp Venous Insufficiency/ or venous insufficiency.mp.	7,970
2	exp Edema/ or edema.mp. or oedema.mp.	136,051
3	exp Non-Filarial Lymphedema/ or exp Lymphedema/ or lymphedema.mp. or lymphoedema.mp. or lymphedemas.mp. or lymphoedemas.mp.	11,775
4	(lymphedemic or lymphoedemic).mp.	4
5	lymphatic.ti,ab.	37,681
6	1 or 2 or 3 or 4 or 5	186,281
7	exp "Quality of Life"/ or quality of life.mp. or QoL.mp. or HRQoL.mp.	215,063
8	life quality.mp.	4,055
9	quality adjusted life.mp.	11,465
10	well being.ti,ab.	43,143
11	health status.mp. or Health Status/	117,736
12	mental health.ti,ab.	83,084
13	daily living.ti,ab.	20,508
14	general health.ti,ab.	18,564
15	physical function.ti,ab.	7,310
16	7 or 8 or 9 or 10 or 11 or 12 or 13 or 14 or 15	431,326
17	conservative treatment.mp.	21,538
18	cdp.mp.	3,619
19	cdt.mp.	1,888
20	decongestive.mp.	293
21	lymphatic drainage.mp.	2,372
22	MLD.mp.	1,910
23	manual lymphatic.mp.	156
24	lymphatic therapy.mp.	108
25	exp drainage/ and exp lymphedema/	318
26	compression therapy.mp.	947
27	pneumatic compression*.mp.	1,187
28	compression garment.mp.	72
29	compression bandage*.mp.	959
30	compression hosiery.mp.	123
31	compression therap*.mp.	955
32	exp Compression Bandages/	1,715
33	((foldi or vodder) and method).mp.	8
34	exp Massage/ or Massage.mp.	11,454
35	physical therap*.mp.	41,317
36	management.mp. and exp lymphedema/	767
37	treatment.mp. and exp lymphedema/	3,162
38	exp Musculoskeletal Manipulations/	14,047
39	17 or 18 or 19 or 20 or 21 or 22 or 23 or 24 or 25 or 26 or 27 or 28 or 29 or 30 or 31 or 32 or 33 or 34 or 35 or 36 or 37 or 38	96,376
40	6 and 16 and 39	556

Database searched on 27.06.2016.

EMBASE search expression

#	Searches	
1	exp vein insufficiency/ or venous insufficiency.mp. or vein insufficiency.mp.	11,343
2	exp edema/ or (edema or oedema).ti,ab.	304,050
3	exp lymphedema/ or (lymphedema* or lymphoedema*).ti,ab.	19,182
4	exp lymph vessel/	29,235
5	lymphatic.ti,ab.	63,055
6	(lymphedemic or lymphoedemic).ti,ab.	7
7	1 or 2 or 3 or 4 or 5 or 6	395,725
8	exp “quality of life”/ or quality of life.ti,ab. Or QoL.mp. or HRQOL.mp.	400,876
9	life quality.ti,ab.	8,467
10	quality adjusted life.ti,ab.	12,043
11	quality adjusted year*.ti,ab.	83
12	exp wellbeing/ or (“well-being” or “well being” or “wellbeing”).ti,ab.	87,480
13	exp health status/	176,797
14	exp mental health/	108,864
15	8 or 9 or 10 or 11 or 12 or 13 or 14	686,762
16	conservative treatment.ti,ab. Or conservative treatment/	81,538
17	cdp.mp.	5,095
18	cdt.mp.	3,172
19	(decongestive and (lymphatic or therapy or physiotherapy)).ti,ab.	504
20	exp lymphatic drainage/	5,679
21	(lymphatic and (drainage* or therap* or manual)).ti,ab.	14,983
22	MLD.mp.	3,583
23	exp compression therapy/	7,324
24	(compression* and (garment or hosiery or bandage* or therap*)).ti,ab.	17,058
25	((foldi or vodder) and method).mp.	25
26	exp massage/ or massage*.ti,ab.	19,326
27	treatment*.mp. and exp lymphedema/	7,221
28	therap*.mp. and exp lymphedema/	5,714
29	physiotherapy.mp. and exp lymphedema/ [mp=title, abstract, heading word, drug trade name, original title, device manufacturer, drug manufacturer, device trade name, keyword]	900
30	16 or 17 or 18 or 19 or 20 or 21 or 22 or 23 or 24 or 25 or 26 or 27 or 28 or 29	156,390
31	7 and 15 and 30	1,813

Database searched on 27.06.2016.

PsycINFO search expression

#	Searches	
1	venous insufficiency.mp.	122
2	exp EDEMA/ or edema.mp. or oedema.mp.	2,734
3	(lymphoedema or lymphedema).mp.	158
4	lymphatic.mp.	1,238
5	1 or 2 or 3 or 4	4,177
6	exp "Quality of Life"/ or quality of life.mp. or QoL.mp. or HRQOL.mp.	58,281
7	life quality.mp.	1,373
8	quality adjusted life.mp.	976
9	exp Mental Health/ or exp Physical Health/ or health status.mp.	66,399
10	well-being.mp. or exp Well Being/	65,009
11	daily living.mp.	15,520
12	physical function.mp.	1,800
13	general health.mp.	12,699
14	6 or 7 or 8 or 9 or 10 or 11 or 12 or 13	191,565
15	cdp.mp.	605
16	cdt.mp.	503
17	decongestive.mp.	11
18	(lymphatic therapy or manual lymphatic or MLD).mp.	319
19	exp Physical Therapy/ or physical therap*.mp.	4,230
20	drainage.mp.	525
21	compression.mp.	2,923
22	exp MASSAGE/ or massage.mp.	1,274
23	exp TREATMENT/ or treatment.mp.	896,259
24	exp MANAGEMENT/ or management.mp.	215,794
25	15 or 16 or 17 or 18 or 19 or 20 or 21 or 22 or 23 or 24	1,040,204
26	5 and 14 and 25	188

Database searched on 27.06.2016.

Cochrane Central Register of Controlled Trials search expression

#	Searches	
1	MeSH descriptor: [Lymphedema] explode all trees	349
2	lymphoedema or "lymphoedemas" or "lymphedemas" or "Lymphoedemas" or "oedema" or "edema" or "oedemas" or "edemas" or "lymphedemic" or "lymphoedemic"	11,327
3	MeSH descriptor: [Edema] explode all trees	1,212
4	MeSH descriptor: [Venous Insufficiency] explode all trees	439
5	venous insufficiency	810
6	1 or 2 or 3 or 4 or 5	12,212
7	quality of life	49,944
8	MeSH descriptor: [Quality of Life] explode all trees	17,782
9	life quality	2,171
10	quality adjusted life	6,966
11	health status or "well being" or "mental health" or "daily living" or "physical function" or "general health"	35,248
12	qol or "HRQoL"	9,266
13	MeSH descriptor: [Health Status] explode all trees	6,169
14	7 or 8 or 9 or 10 or 11 or 12 or 13	79,585
15	drainage	5,614
16	cdp	250
17	cdt	159
18	decongestive	145
19	drain*	6,794
20	mld or "manual lymphatic" or "manual therapy" or "manual therapies"	1,133
21	lymph* therap*	20,027
22	compression	5,557
23	("foldi" or "vodder") and "method"	8
24	MeSH descriptor: [Massage] explode all trees	884
25	massage	2,790
26	treatment or "treatments" or "management" or "physical" or "intervention"	537,484
27	MeSH descriptor: [Physical Therapy Modalities] explode all trees	18,743
28	15 or 16 or 17 or 18 or 19 or 20 or 21 or 22 or 23 or 24 or 25 or 26 or 27	550,505
29	6 and 14 and 28 (Trials)	734

Database searched on 27.06.2016.

Cochrane database of systematic reviews search expression

#	Searches	
1	manual lymphatic drainage	99
2	complete decongestive therapy	15
3	complete decongestive physiotherapy	7
4	complex decongestive therapy	21
5	decongestive lymphatic therapy	23
6	1 or 2 or 3 or 4 or 5	128
7	quality of life	57,544
8	6 and 7 (Filter Reviews)	19

Database searched on 27.06.2016.

ClinicalTrials.gov search expression

#	Searches	
1	Outcome: Quality of life OR QOL OR HRQOL OR Well Being OR life quality OR quality adjusted life OR daily living OR physical function OR general health	28,311
2	venous insufficiency OR edema OR oedema OR lymphedema OR lymphoedema OR lymphedemas OR lymphoedemas lymphedemic OR lymphoedemic OR lymphatic	6,634
3	INFLECT EXACT NOT ("Recruiting" OR "Not yet recruiting" OR "Available") [OVERALL-STATUS] AND NOT NOTEXT [FIRST-RECEIVED-RESULTS-DATE] AND INFLECT EXACT "Interventional" [STUDY-TYPES] AND (venous insufficiency OR edema OR oedema OR lymphedema OR lymphoedema OR lymphedemas OR lymphoedemas lymphedemic OR lymphoedemic OR lymphatic) [DISEASE] AND (Quality of life OR QOL OR HRQOL OR Well Being OR life quality OR quality adjusted life OR daily living OR physical function OR general health) [OUTCOME]	146

Database searched on 27.06.2016.
