# Supplementary material for: Manual lymphatic drainage and quality of life in patients with lymphoedema and mixed oedema: a systematic review of randomised controlled trials
Source: Qual Life Res. 2018 Feb 5;27(6):1403–14. doi: 10.1007/s11136-018-1796-5 (PMC5951867; doi:10.1007/s11136-018-1796-5)
Supplement: Supplementary file 2 — Supplementary material 2 (PDF 71 KB) [file 11136_2018_1796_MOESM2_ESM.pdf]

Appendix S2. Overall HRQoL reported for each arm of the RCTs at baseline and post-intervention, and statistical significance of the effect of the interventions within each group and between groups. The scores are given as mean (SD) or median (range).

First author (year of publication)	Aetiology, area with oedema	HRQoL measurement				Intervention group (IG)			Control group (CG)			IG vs. CG
		Scale	Disease specific	Oedema specific	Interpretation	Baseline	Follow-up	p-value[1]	Baseline	Follow-Up	p-value[1]	p-value[2]
Belmonte (2012) [26]	BCRL, arm	FACT-G	No	No	Higher score indicates better status (Range: 0-100)	69.7 (11.9)[3]	69.9 (14.1)[3]	n.r.	74.3 (13.5)[3]	77.2 (14.0)[3]	n.r.	**0.13**[4]
		FACT-B	Yes	No	Higher score indicates better status (Range 0-144)	91.4 (18.4)[3]	88.9 (17.0)[3]	**n.r.**	96.7 (16.7)[3]	99.6 (19.0)[3]	**n.r.**	**0.08**[4]
		FACT-TOI	Yes	No	Higher score indicates better status (Range 0-84)	60.3 (12.3)[3]	55.9 (12.4)[3]	**n.r.**	59.9 (13.0)[3]	61.9 (13.6)[3]	**n.r.**	**0.17**[4]
Gradalski (2015) [29]	BCRL, arm	QoL-LQ	No	Yes	Lower score indicates better status (Range: 0-10)	3.1 (1.5)	1.4 (1.1)	**<0.001**	3.2 (1.7)	1.8 (1.1)	**<0.001**	**0.4**
Odebiyi (2014) [31]	BCRL, arm	EORTC QLQ-C30	No	No	Lower score indicates better status (Range: 0-126)	84.2 (22.0)	49.4 (9.9)	**<0.001**	91.1 (13.7)	79.5 (12.3)	**0.06**	**<0.001**
Ridner (2013) [32]	BCRL, arm	ULL-27	No	Yes	Higher score indicates better status (Range: 0-100)	69 (52-88)	79 (62-91)	**n.r.**	80 (65-91)	90 (54-98)	**n.r.**	**0.59**[5]
		FACT-G	No	No	Higher score indicates better status (Range: 0-100)	91 (32-107)	86 (60-105)	**n.r.**	88 (32-107)	92 (50-105)	**n.r.**	**0.32**[5]
		FACT- B	Yes	No	Higher score indicates better status (Range 0-144)	116 (64-133)	110 (91-136)	**n.r.**	111 (40-134)	114 (62-134)	**n.r.**	**0.25**[5]
Bongi (2011) [27]	SSc, hand	HAQ-DI	No	No	Lower score indicates better status (Range: 0-3)	1.6 (0.8)	0.9 (0.7)	**<0.001**	1.5 (0.9)	1.5 (0.9)	**>0.05**	**0.05**
Holmes (2014) [30]	PTS, leg	VEINES-QOL	Yes	No	Higher score indicates better status	51 (7)	50 (6)	**0.17**	49 (6)	50 (7)	**0.84**	**0.43**
dos Santos Crisostomo (2015) [33]	CVI, leg	CIVIQ-20	Yes	No	Lower score indicates better status (Range: 0-100)	48.1 (12.5)	44.8 (14.9)	**n.r.**	48.7 (16.0)	50.2 (17.6)	**n.r.**	**>0.05**

Notes

Results for Dayes et al, 2013 were presented by domain only and therefore not presented here.

[1]p-value for the comparison of the mean scores obtained pre- and post-intervention within each arm of the trial. All studies used a significance level of p=0.05.

[2]p-value for the comparison of the mean scores between the IG and CG. All studies used a significance level of p=0.05.

[3]cross-over trial: data extracted only for the first cycle to avoid carry over and period effects.
[4]p-value calculated using the information given in the original study using an independent sample t-test.
[5]p-value of the mixed general linear modeling analysis. The two factors included in this analysis were group (LLLT, MLD, and MLD and LLLT) and time of assessment (baseline, last treatment).

Abbreviations

CIVIQ-20, Chronic Venous Insufficiency Quality of Life Questionnaire-20;
CG, control group;
EORTC QLQ-C30, European Organization for Research and Treatment of Cancer Quality of Life Questionnaire core 30;
FACT-B, FACT-G, FACT-TOI, Functional Assessment of Cancer Therapy(-breast)(-general)(-trial outcome index);
HAQ-DI, Health Assessment Questionnaire for Rheumatoid Arthritis;
HRQoL, Health-related Quality of Life;
IG, intervention group;
LLLT, Low-level laser therapy;
MLD, manual lymphatic drainage;
n.r., not reported;
QoL-LQ, Quality-of-life Lymphoedema Questionnaire;
ULL-27, Upper Limb Lymphoedema-27;
VEINES-QOL, Venous Insufficiency Epidemiological and Economic Study Quality of Life questionnaire.
