# Supplementary material for: Manual lymphatic drainage and quality of life in patients with lymphoedema and mixed oedema: a systematic review of randomised controlled trials
Source: Qual Life Res. 2018 Feb 5;27(6):1403–14. doi: 10.1007/s11136-018-1796-5 (PMC5951867; doi:10.1007/s11136-018-1796-5)
Supplement: Supplementary file 3 — Supplementary material 3 (PDF 107 KB) [file 11136_2018_1796_MOESM3_ESM.pdf]

Appendix S3. HRQoL domains reported for each arm of the RCTs at baseline and post-intervention, and statistical significance of the effect of the interventions within each group and between groups. The scores are given as mean (SD) or median (range).

HRQoL domain				Intervention group (IG)			Control group (CG)			IG vs. CG
	Aetiology, area with oedema	Scale	Condition specific instrument	Baseline score	Follow-up score	p-value[1]	Baseline score	Follow-up score	p-value[1]	p-value[2]
**Physical functioning**										
Belmonte (2012) [26]	BCRL, arm	FACT-B Physical well-being subscale[3]		22.7 (3.6)	22.0 (4.5)	n.r.	21.5 (6.2)	22.6 (6.2)	n.r.	0.245[7]
		VAS tightness[3]		33.8 (27.7)	33.0 (28.0)	n.r.	20.9 (27.2)	13.3 (24.6)	n.r.	0.049[7]
		VAS heaviness[3]		37.5 (34.1)	34.7 (34.4)	n.r.	37.2 (34.5)	22.4 (27.4)	n.r.	0.673[7]
Dayes (2013) [28]	BCRL, arm	SF-36 PSI		42.7 (6.7)	43.9 (7.8)	>0.1	42.6 (7.4)	43.8 (7.1)	>0.1	>0.1
		DASH		32.2 (21.8)	32.3 (21.5)	>0.1	32.2 (23.8)	29.7 (22.4)	>0.1	>0.1
Gradalski (2015) [29]	BCRL, arm	Limb heaviness[4]	X	4.0 (2.6)	1.6 (2.3)	0.0017	4.0 (3.3)	1.9 (1.8)	0.008	0.7
		Movement Restriction[4]	X	3.1 (3.0)	1.6 (2.4)	0.06	3.1 (3.8)	1.7 (2.1)	0.1	0.7
Bongi (2011) [27]	SSc, hand	SF-36 PSI		38.8 (7.9)	44.8 (8.3)	<0.001	39.2 (7.0)	39.0 (7.4)	>0.05	<0.05
Holmes (2014) [30]	PTS, leg	Leg heaviness[6]	X	2.3 (1.0)	2.0 (1.0)	0.14	2.4 (1.0)	1.9 (1.0)	0.05	0.783[7]
dos Santos Crisostomo (2015) [33]	CVI, leg	CIVIQ-20 physical		54.0 (16.3)	48.7 (19.9)	n.r.	50.2 (21.2)	50.7 (21.2)	n.r.	>0.05
		Heaviness (VAS)		5.3 (2.4)	2.1 (2.2)	0.001	4.7 (2.9)	4.3 (2.9)	>0.05	0.001
**Role functioning**										
Belmonte (2012) [26]	BCRL, arm	FACT-B Functional well-being subscale[3]		16.3 (3.7)	15.2 (6.7)	n.r.	15.3 (5.7)	15.8 (5.0)	n.r.	0.109[7]
Gradalski (2015) [29]	BCRL, arm	Interference with work[4]	X	0.9 (2.0)	0.4 (1.1)	0.3	0.6 (2.1)	0.5 (1.7)	0.9	0.3
		Issues with clothing[4]	X	5.3 (3.0)	2.2 (2.2)	<0.001	5.9 (3.0)	3.1 (2.8)	0.001	0.7
		Interference with household[5]	X	4.3 (3.1)	3.3 (2.3)	0.2	5.1 (3.1)	3.9 (2.6)	0.2	0.9
		Dependence others	X	2.3 (2.7)	2.2 (2.7)	0.9	3.4 (3.4)	2.5 (2.7)	0.3	0.6
Bongi (2011) [27]	SSc, hand	Inference life (VAS)	X	4.3 (2.5)	2.0 (1.8)	<0.001	4.5 (2.5)	4.1 (2.3)	>0.05	<0.01
**Psychological functioning**										
Belmonte (2012) [26]	BCRL, arm	FACT-B Emotional well-being subscale[3]		13.9 (5.6)	14.8 (5.5)	n.r.	18.1 (4.4)	18.4 (4.0)	n.r.	0.140[7]
Dayes (2013) [28]	BCRL, arm	SF-36 MSI		42.4 (6.3)	44.3 (7.0)	>0.1	44.2 (5.6)	44.3 (5.8)	>0.1	>0.1
Gradalski (2015) [29]	BCRL, arm	Sleep disturbance[4]	X	3.4 (2.9)	1.1 (2.2)	0.004	2.9 (3.1)	1.4 (2.4)	0.06	0.2
		Inference well being[4]	X	3.7 (2.9)	1.4 (1.9)	0.002	3.9 (3.0)	2.2 (2.2)	0.03	0.4
		Subjective worsening of limb appearance[4]		4.8 (2.8)	1.2 (1.8)	<0.001	5.3 (2.8)	2.0 (2.4)	<0.001	0.6

HRQoL domain				Intervention group (IG)			Control group (CG)			IG vs. CG
	Aetiology, area with oedema	Scale	Condition specific instrument	Baseline score	Follow-up score	p-value[1]	Baseline score	Follow-up score	p-value[1]	p-value[2]
Ridner (2013) [32][5]	BCRL, arm	CES-D		12 (9-38)	11 (8-22)	n.r.	12 (7-20)	12 (7-23)	n.r.	0.985[8]
		POMS		35 (24-88)	31 (24-49)	n.r.	29 (24-111)	29 (24-112)	n.r.	0.878[8]
Bongi (2011) [27]	SSc, hand	SF-36 MSI		38.2 (6.7)	44.4 (6.8)	<0.001	37.2 (6.4)	38.8 (6.5)	>0.05	0.01
dos Santos Crisostomo (2015) [33]	CVI, leg	CIVIQ-20 psychological scale		37.2 (11.1)	42.2 (41.4)	n.r.	39.9 (15.5)	48.3 (18.5)	n.r.	>0.05
**Social functioning**										
Belmonte (2012) [26]	BCRL, arm	FACT-B Social well-being subscale[3]		16.8 (7.3)	17.6 (8.7)	n.r.	19.0 (5.9)	19.5 (4.9)	n.r.	0.440[7]
Gradalski (2015) [29]	BCRL, arm	Interference social life[4]	X	1.8 (2.6)	0.1 (0.4)	0.003	1.5 (3.1)	0.7 (1.6)	0.3	0.1
		Interference intimate relations[4]	X	0.8 (2.2)	0.2 (1.0)	0.2	0.00 (0.00)	0.00 (0.00)	1.0	0.04
		Interference hobbies[4]	X	2.8 (2.8)	0.9 (1.7)	0.007	1.6 (2.8)	1.3 (2.2)	0.7	0.1
dos Santos Crisostomo (2015) [33]	CVI, leg	CIVIQ-20-S		52.0 (19.8)	44.6 (18.2)	n.r.	47.9 (18.6)	49.2 (19.5)	n.r.	>0.05
**Pain** (in the area affected by oedema)										
Belmonte (2012) [26]	BCRL, arm	VAS[3]	X	19.8 (31.1)	22.5 (29.1)	n.r.	16.2	8.4 (21.1)	n.r.	0.182[7]
Gradalski (2015) [29]	BCRL, arm	QoL-LQ-S	X	2.4 (2.3)	1.1 (1.8)	0.04	3.6 (3.0)	1.8 (1.9)	0.01	0.4
Bongi (2011) [27]	SSc, hand	VAS	X	3.8 (2.6)	1.5 (2.1)	<0.001	3.6 (2.8)	3.8 (2.4)	>0.05	<0.01
Holmes (2014) [30]	PTS, leg	Villalta score[6]	X	2.1 (1.1)	1.8 (0.8)	0.06	2.3 (1.2)	1.9 (1.9)	0.17	0.852[7]
dos Santos Crisostomo (2015) [33]	CVI, leg	CIVIQ-20 pain dimension	X	55.8 (14.2)	47.1 (14.2)	0.045	55.7 (16.2)	55.0 (17.6)	n.r.	0.035
**Vitality/Fatigue**										
Odebiyi (2014) [31]	BCRL, arm	EFS		9.1 (5.0)	16.7 (3.2)	<0.001	5.7 (1.0)	6.8 (1.6)	0.02	0.001
Ridner (2013) [32]	BCRL, arm	BFI		1.2 (0-8)	1.6 (0-6)	n.r.	1.3 (0-10)	1.2 (0-7)	n.r.	0.748[8]
dos Santos Crisostomo (2015) [33]	CVI, leg	VAS		5.2 (2.4)	2.2 (2.2)	0.001	4.7 (2.9)	3.9 (2.9)	n.r.	0.012
**General health**										
Ridner (2013) [32]	BCRL, arm	FACT-G		91 (32-107)	86 (60-105)	n.r.	88 (32-107)	92 (50-105)	n.r.	0.319[8]

Notes

[1]p-value for the comparison of the mean scores obtained pre- and post-intervention within each arm of the trial. All studies used a significance level of p=0.05.

[2]p-value for the comparison of the mean scores between the IG and CG. All studies used a significance level of p=0.05.

[3]cross-over trial: data extracted only for the first cycle to avoid carry over and period effects.

[4]QoL-LQ-S: item from the Quality-of-life Lymphoedema Questionnaire.
[5]Data for physical, and role and psychological functioning domains were also assessed but presented in the original publication as effect sizes only, and therefore not extracted.
[6]Post-thrombotic syndrome severity score.
[7]p-value calculated using the information given in the original study using an independent sample t-test.
[8]p-value of the mixed general linear modeling analysis. The two factors included in this analysis were group (LLLT, MLD, and MLD and LLLT) and time of assessment (baseline, last treatment).

Abbreviations
BCR, Breast Cancer Related;
BFI, Brief Fatigue Inventory;
BG, Between-Group difference;
CG, Control Group;
CES-D, Center for Epidemiologic Studies – Depression scale;
CIVIQ-20, Chronic Venous Insufficiency Quality of Life Questionnaire – 20 items;
CVI, Chronic Venous Insufficiency;
DASH, Disabilities of the Arm, Shoulder and Hand scale;
EORTC QLQ-C30, European Organization for Research and Treatment of Cancer Quality of Life Questionnaire Core 30;
EFS, Energy/Fatigue Scale;
FACT-B, FACT-G, FACT-S, Functional Assessment of Cancer Therapy(-breast)(-general)(-subscale);
HAMIS, Hand Mobility Test in Sclerodermia;
HAQ, Health Assessment Questionnaire;
HRQoL, Health-Related Quality of Life;
IG, intervention Group;
LSIDS-A, Lymphoedema Symptom Intensity and Distress Scale - Arm;
MLD, Manual Lymphatic Drainage;
POMS, Profile of Mood States – Short Form;
PP, Pre- vs. Post-intervention mean in the IG;
PTS, Post Thrombotic Syndrome;
QoL-LQ, Quality-of-life Lymphoedema Questionnaire;
SF-36 MSI, PSI, short form-36 mental synthetic index, physical synthetic index;
SSc, Systemic Sclerosis;
-S, -Subscale;
ULL-27, Upper Limb Lymphoedema-27;
VAS, Visual Analog Scale;

VCSS, Venous Clinical Severity Score;
VEINES-QOL, Venous Insufficiency Epidemiological and Economic Study Quality of Life questionnaire.
